# The Effect of Ceftriaxone in Valproic Acid-Induced Mouse Model of Autism

**DOI:** 10.34172/apb.2022.086

**Published:** 2021-10-06

**Authors:** Gamze Gur, Ruhan Deniz Topuz, Gulnur Kizilay

**Affiliations:** ^1^Department of Medical Pharmacology, Faculty of Medicine, Trakya University, 22030-Edirne, Turkey.; ^2^Department of Histology Embryology, Faculty of Medicine, Trakya University, 22030-Edirne, Turkey.

**Keywords:** Autism spectrum disorders, Ceftriaxone, GLT-1, Valproic acid

## Abstract

***Purpose:*** Autism is a multifactorial neurodevelopment disease and it has not been disclosed as a hypoglutamatergic or hyperglutamathergic disease. Ceftriaxone is an antibiotic that increases glutamate transporter-1 (GLT-1) expression in the brain in chronic use. In our study we aimed to investigate the effects of different doses of ceftriaxone in postnatal period in male mice exposed to valproic acid (VPA) at 12.5th day of pregnancy.

***Methods:*** A total of 96 BALB/c male mice were divided into 12 groups (n = 8 animals per group). Ceftriaxone (50, 100, 200 mg/kg/d) or saline was given to the male offsprings born from pregnant mice administered VPA and/or saline, between days 47 and 55. Dihydrokainic acid (10 mg/kg), a GLT-1 inhibitor, was administered intraperitoneally to evaluate whether GLT-1 mediates the effect of ceftriaxone. Three chamber sociability and social interaction test and the rota rod test were performed in all groups on days 54 and 55. GLT-1 levels in the hippocampus were measured by immunohistochemistry (IHC) and western blotting (WB).

*** Results:*** In our study, autism-like behaviors were observed in male offsprings that were exposed to VPA in the intrauterine period. Chronic ceftriaxone administration has no curative effect on behavioral impairment seen in autism.

***Conclusion:*** Our results show that ceftriaxone did not exert significant therapeutic effect on VPA-induced mouse model of autism.

## Introduction

Autism spectrum disorder (ASD) is a common and serious neurodevelopmental disorder characterized by two main symptoms; lack of social communication skills and repetitive behavior. Patients may exhibit various symptoms such as stereotyped or repetitive movements or speech, reduced sharing of interests, emotions or affect, abnormalities in eye contact, insistence on sameness and inflexible adherence to routines.^1,2^ Since ASD is a multi- symptomatic disease, it is difficult to diagnose.

The etiology of autism has not been fully elucidated. Prenatal exposure to viruses, toxic substances and various pathogens may cause ASD. Likewise, gestational diabetes, maternal infections (rubella, cytomegalovirus, etc), maternal inflammation and drugs such as valproic acid (VPA), thalidomide, misoprostol, heavy metals and ethanol may be associated with ASD.^3,4^

Central nervous system develops in between embryonic days 7.5 and 18.5 in mice.^5^ Autism-like behaviors are reported in mice exposed to different doses of VPA during this period. Offsprings whose mothers received a single dose of 600 mg/kg VPA injection at day 12.5 of gestation are particularly considered to be autistic and used in experimental studies.^6^ It is not known exactly how VPA causes autistic behaviors. Determination of folic acid metabolism, inhibition of histone deacetylation and increased oxidative stress may play roles in this process.^6-8^

The pathophysiology of autism is unclear and unbalance of GABA and glutamate ratios may cause ASD.^9^ In autistic patients, it has been shown that plasma GABA levels are higher and glutamate/GABA ratio is lower.^10^ Although there are studies reporting the contrary.^11^ Glutamate is the most important excitatory neurotransmitter in the brain, and glutamate transporters regulate extracellular glutamate homeostasis. Glutamate transporter-1 (GLT-1) is responsible for 90% of glutamate uptake and organizes synaptic transmission of glutamate.^12^ Beta-lactam antibiotics stimulate GLT-1 expression by increasing the transcription of the *GLT-1* gene.^13,14^ It has been shown that when beta lactam antibiotic, ceftriaxone, administered to animals; expression of GLT-1 in brain and its biochemical and functional activity were increased. Also it exerts neuroprotective effects by stimulating GLT-1 expression.^13^ In addition we showed that chronic ceftriaxone injection increased GLT-1 expression in hippocampus in mice.^15^

 Based on this information we aimed to investigate the effect of ceftriaxone in male offspring exposed to VPA in the embryonic period and determine whether ceftriaxone-induced increase in GLT-1 levels in the brain has any effect on behavioral impairment in ASD.

## Material and Methods

###  Animals 

Male and female BALB/c mice, weighing 20–30 g at the beginning of the experiments, were used in this project. Animals were housed in a quiet room, and water and food were provided *ad libitum*.

###  Drugs and experimental procedures

 Mice were mated for 48 hours, and the presence of a vaginal plug was considered as gestational day 0. Pregnant mice received a single intraperitoneal (i.p.) injection of 600 mg/kg VPA (Sigma-Aldrich, China) or saline on gestational day 12.5. Only male offspring born from mothers exposed to saline or VPA were used. The male offspring were separated from the dams at 21st day of life and the rota rod test, three chamber sociability and social interaction test (3 CHM) were performed on postnatal day 54-55.

 Ceftriaxone (50–200 mg/kg, i.p.) was administered on postnatal days 47-55 for 8 days. Ceftriaxone (Rocephin, Roche) was diluted from commercial preparations and dissolved in saline. Dihydrokainic acid 10 mg/kg (Sigma Aldrich, USA), a GLT-1 blocker, was co-administered with ceftriaxone 200 mg/kg group. All drugs and saline were administered i.p. in a volume of 0.1 mL/10 g body weight.

 After having completed all behavioral tests, mice were euthanized and brain tissues were dissected for western blotting (WB) and immunohistochemistry (IHC) analysis.

###  Behavioral Tests

####  Three chamber sociability and social interaction test 

Three chamber sociability and social interaction test (3 CHM) was performed with minimal modification as previously reported.^16^ The test apparatus consists of a plexiglas box with a total size of 60 × 40 × 20 cm divided into three chambers. There are openings between the compartments, that allow exploring of all the arena and two wire cages. The test was performed on two days; on first day, mice was free in three chamber boxes for 30 minutes for habituation. On the second day the task consisted of three sessions. The first session was the habituation period for 5 minutes. Subject animal was left in the center area and allowed to roam freely. After habituation, the second session (sociability test) lasted for 10 minutes and a novel animal was introduced in the wire cage of the left compartment. In this part, the time spent near the full /empty cage and sniffing duration of the wire cages were calculated. In the last 10 minutes, the animal in the cage of the left compartment (familiar animal) was resettled in the wire cage of the right compartment and a new novel animal (unfamiliar animal) was placed into the wire cage of the left compartment. The time spent in compartments and the sniffing duration of the familiar/unfamiliar animals were calculated in the last part for evaluation of social novelty. All records were analyzed using EthoVision XT 11.5 (Noldus, The Netherlands), Mouse Behaviour Module.

####  Rota rod performance test

 A rota rod apparatus (Commat, Ankara, Turkey) was used to evaluate locomotor coordination. The mouse was left on a rotating bar and the time to fall from the bar was recorded. The cut-off time was set to 180 seconds.

###  Western blotting 

Western blot analyses were performed as described previously.^17^ Briefly, samples were loaded into NuPAGE Novex 4–12% Bis-Tris gel (Life Technologies, Invitrogen, Carlsbad, CA, USA), electrophoretically separated and electroblotted onto a nitrocellulose membrane (The iBlot^TM^ Gel Transfer Stack, Nitrocellulose, Invitrogen). The blocking solution was used for blocking membrane for 30 minutes to reduce nonspecific binding (Western Breeze Chemiluminescent Western Blot Immunodetection Kit Antirabbit; Invitrogen). Subsequently, the membranes were incubated overnight at 4°C with rabbit polyclonal EAAT2/GLT1 (1:1000 dilution in blocking solution, Novus Biologicals, Littleton, CO). The membrane was washed two times for 10 minutes with wash buffer and then incubated with secondary antibody for 30 minutes (Western Breeze Chemiluminescent Western Blot Immunodetection Kit Antirabbit; Invitrogen). Following washing three times for 10 minutes with wash buffer, the protein was visualized by ChemiDoc^TM^ MP Imaging System (Universal Hood 3; Bio-Rad, Hercules, CA, USA) with enhanced chemiluminescence substrate (Invitrogen). Then, the same membranes were washed according to the protocol using Restore Western Blot Stripping Buffer (Thermo Fisher Scientific, Fremont, CA). The same membranes were then incubated with rabbit polyclonal β-actin Antibody (Novus Biologicals) at a dilution of 1/1000 in a shaker overnight at + 4ºC. The procedures for GLT-1 were repeated in the same order and imaging was performed. Immunoblot bands for GLT-1 and β-actin were quantified using an ImageJ 1.48v program (Wayne Rasband, National Institutes of Health, Bethesda, MD, USA). The ratio of the density of GLT-1 to β-actin was calculated for each sample.

###  Immunohistochemistry

For immunohistochemical examinations, 5 µm thick sections were taken from paraffin-embedded brain tissue samples and incubated overnight in a 56°C oven. Subsequently, deparaffinization with toluene (Merck Millipore, Darmstadt, Germany) and rehydration with graded series of ethanol (70-100%) were boiled in citrate buffer (pH 6, Invitrogen) for antigen recovery. The sections were then exposed to H_2_O (Abcam, Cambridge, USA) to remove endogenous peroxidase activity. In order to prevent nonspecific binding, the blocking antibody (Invitrogen) was used for 10 minutes. The incubated sections were incubated overnight at + 4°C in EAAT2 / GLT1 antibody (Novus Biologicals) prepared with antibody dilution solution (Invitrogen) at room temperature. Biotinylated secondary antibody (Invitrogen) against the species from which the primary antibody is produced is administered for 10 minutes. and finally treated with HRP-streptavidin (Invitrogen) for 10 minutes. Staining reactions were visualized using DAB (Invitrogen), then counterstained with hematoxylin. Sections were examined using a ×200 objective on a BX-51 Olympus microscope (Olympus, Tokyo, Japan) and photographed. EAAT2/GLT1 immunoreactivity were semi-quantitatively evaluated using a histological score (HSCORE) value, average score was used for statistical analysis, described previously.^17^

###  Statistical analysis

Three chamber sociability and social interaction test, the rota rod test, immunohistochemical examination and WB results were analyzed using *t* test in two groups comparisons, while one-way variance analysis (ANOVA) and post hoc Bonferroni test was used in multiple comparisons. The analyzes were performed on GraphPad Prism 6.0 for Mac OS X software and *P* ˂ 0.05 was considered significant.

## Results

 In this study, the effects of ceftriaxone (50, 100, 200 mg/kg) were evaluated on the VPA-induced mouse model of autism. In addition, changes in GLT-1 levels in the hippocampus were evaluated by IHC and WB analysis. Rota rod test, three chamber sociability and social interaction test were performed in all groups on days 54 and 55. The data obtained from the study will be presented in four sections:

###  Effect of prenatal VPA administration

**Figure 1:**
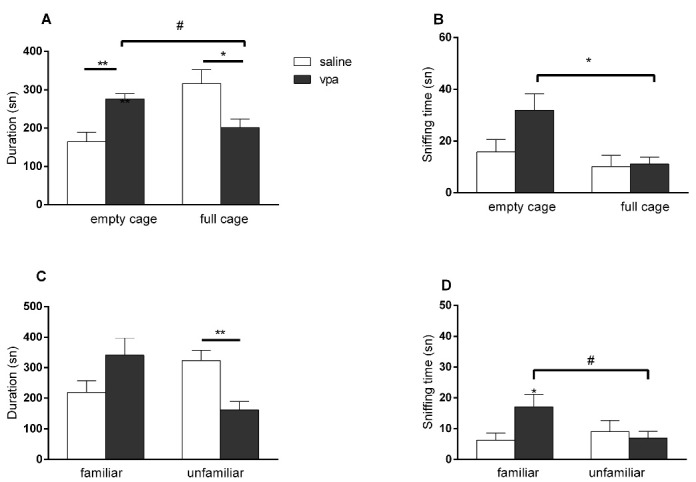
Effects of intrauterine VPA injection in three chamber sociability and social interaction test. (A) Time spent near the empty/full cage; (B) Total sniffing duration of the empty/full cage; (C) Time spent near the familiar/unfamiliar animal; (D) Total sniffing duration of the familiar/unfamiliar animal. (*P < 0.05; ** P < 0.01 vs saline exposed offsprings, unpaired t test, # *P* <0.05 vs same group, paired t test. Vertical lines indicate SEM)

In the VPA exposed offsprings the time spent near the full cage was significantly lower than the saline group (*P* = 0.0229; unpaired *t* test), but no difference was observed in the sniffing times of empty or full cages (*P* = 0.2959; unpaired *t* test) in the sociability phase of 3 CHM test. In VPA exposed group, sniffing time of the full cage was also lower compared to sniffing time of empty cage (*P* = 0.0324; paired *t* test) (Figure 1A and 1B). In the social interaction phase the time spent near the unfamiliar animal (*P* = 0.0375; unpaired *t* test) and the sniffing time of the unfamiliar animal (*P* = 0.0335; unpaired *t* test) were lower in the VPA exposed offsprings compared to saline groups; sniffing duration of the unfamiliar animal was also lower compared to familiar animal in VPA exposed group (*P* = 0.0243; paired *t* test) (Figure 1C and 1D). There were no differences between the groups in the rota rod test (data not shown).

###  Effects of ceftriaxone on intrauterine saline exposed offsprings

There were no differences in the time spent near full/ empty cage among groups in the first part of the 3 CHM. In this stage of the experiment, the time spent near the full cage were higher than the empty cage in the 50 mg/kg and 100 mg/kg cef groups. However, ceftriaxone decreased sniffing times of the empty cage at all doses used (*P* = 0.0003; ANOVA). In the second part of the test ceftriaxone had no effect in any parameter, however the time spent with the unfamiliar mice was greater than the time spent with the familiar mice in the 50 mg/kg cef group (*P* = 0.0108 paired *t* test). The time spent on rod was not different among groups (data not shown).

###  Effects of postnatal ceftriaxone treatment on intrauterine VPA exposed offsprings

**Figure 2:**
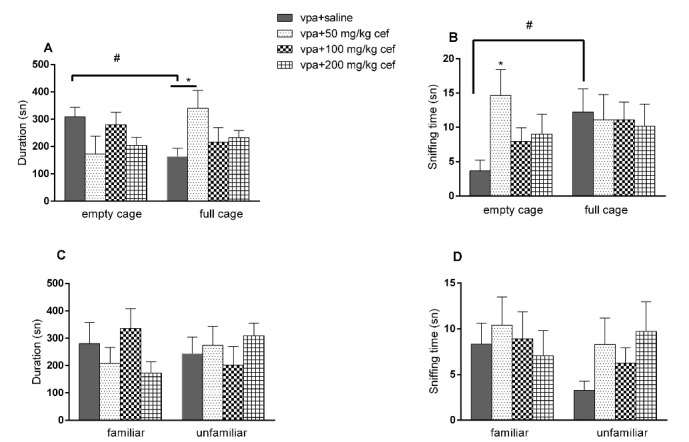
Effects of ceftriaxone on VPA exposed offsprings in three chamber sociability and social interaction test. (A) Time spent near the empty/full cage; (B) Total sniffing duration of the empty/full cage; (C) Time spent near the familiar/unfamiliar animal; (D) Total sniffing duration of the familiar/unfamiliar animal. (*P < 0.05; vs control, one-way ANOVA. #* P* <0.05 vs same group, paired t test. Vertical lines indicate SEM)

In the sociability phase of 3 CHM test, there were no difference in the time spent near the full/empty cages, but sniffing times of the empty cage was increased in 50 mg/kg ceftriaxone group (*P*= 0.0506; ANOVA). There was also a difference in terms of the time spent in the near full/empty cage in the control group (*P* = 0.0487; paired *t* test) (Figure 2A and 2B). In the social novelty phase, there were no significant differences in the time spent near the full/empty cage or sniffing time of the familiar/unfamiliar animals at any doses of ceftriaxone compared to saline group (Figure 2C and 2D). Ceftriaxone had no effect in the time spent on rod in the rota rod test (data not shown).

###  Effects of postnatal dihydrokainic acid injection on intrauterine VPA exposed offsprings

**Figure 3:**
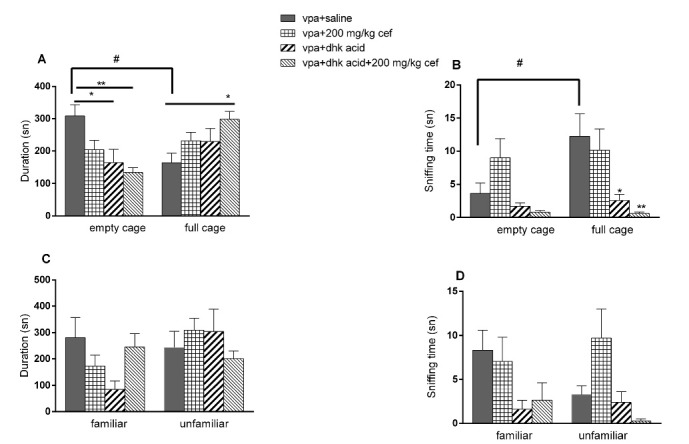
Effects of dihydrokainic acid on VPA exposed offsprings in three chamber sociability and social interaction test. (A) Time spent near the empty/full cage; (B) Total sniffing duration of the empty/full cage; (C) Time spent near the familiar/unfamiliar animal; (D) Total sniffing duration of the familiar/unfamiliar animal. (*P < 0.05; ** P < 0.01 vs control, one-way ANOVA. Vertical lines indicate SEM)

There was an increase in the dihydrokainic acid + 200 mg/kg cef group in terms of the time spent near the full cage (Figure 3A and 3B, *P* = 0.0304, ANOVA) In the second part of 3 CHM, no difference was observed in any parameters (Figure 3C and 3D).

###  Western blotting and immunohistochemistry results

**Figure 4:**
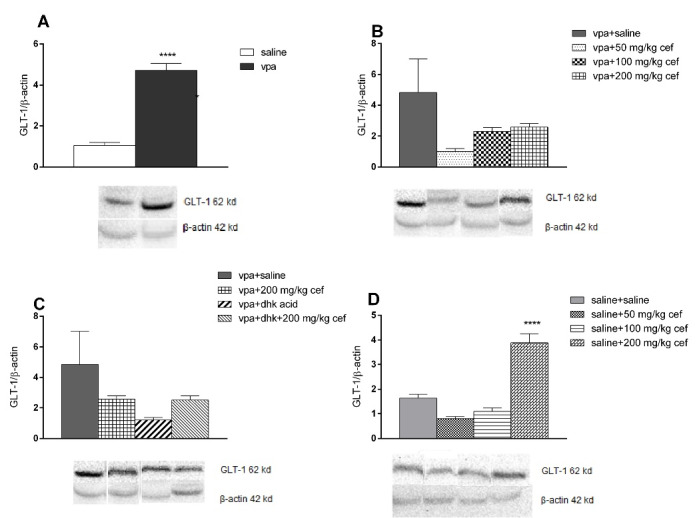
Levels of GLT-1 in western blot. (**** *P* <0.0001 vs control unpaired t test, vertical lines indicate SEM)

**Figure 5:**
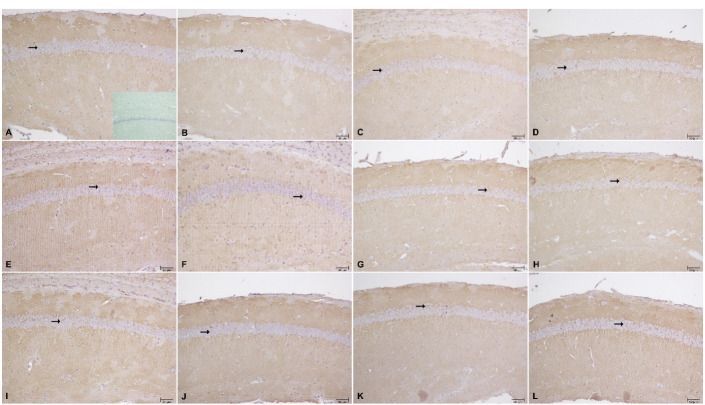
Immunoreactivity of GLT-1 in CA1 of hippocampus: (A) Saline exposed group, inset photograph negative control, (B) Vpa exposed group, (C) Vpa exposed + Saline treatment, (D) Vpa exposed+50 mg/kg cef, (E) Vpa exposed+100 mg/kg cef, (F) Vpa exposed+200 mg/kg cef, (G) Vpa exposed + DHC acid, H) Vpa exposed + DHC acid+200 mg/kg cef, (I) Saline exposed + saline, (J) Saline exposed+50 mg/kg cef, (K) Saline exposed+100 mg/kg cef, (L) Saline exposed+200 mg/kg cef (scale bar 50 μm, magnification ×200)

GLT-1 levels were higher in the VPA exposed offsprings than the saline exposed groups in WB (*P*<0.0001; unpaired t test), but there were no differences between these groups in IHC (Table 1, Figure 4A and Figure 5).

**Table 1:** Immunoreactivity of GLT-1 in all groups (mean + SD)

**Groups**	**GLT-1 immunoreactivity**
Saline (A)	160.0 ± 15.58
Vpa (B)	155.0 ± 15.21
Vpa+Saline (C)	175.6 ± 7.763
Vpa+50 mg/kg cef (D)	156.9 ± 18.50
Vpa+100 mg/kg cef (E)	175.6 ± 30.76
Vpa+200 mg/kg cef (F)	216.9 ± 26.04
Vpa+dhc acid (G)	187.9 ± 11.85
Vpa+dhc acid+200 mg/kg cef (H)	192.5 ± 16.04
Saline+saline (I)	186.9 ± 22.19
Saline+50 mg/kg cef (J)	158.1 ± 20.69
Saline+100 mg/kg cef (K)	191.9 ± 27.25
Saline+200 mg/kg cef (L)	191.3 ± 18.08

In WB, GLT-1 level was higher in 200 mg/kg chronic ceftriaxone group compared to control in saline exposed offspring (*P* <0.0001; ANOVA) (Figure 5D); however, there were no differences in IHC findings (Table 1 and Figure 5).

GLT-1 level did not change in VPA exposed offsprings in WB (Figure 5B), but were higher than control groups in 200 mg/kg ceftriaxone group in IHC (*P* = 0.0001; ANOVA) (Table 1 and Figure 5).

WB (Figure 4C) and IHC data (Table 1 and Figure 5) show that dihydrokainic acid had no effect on GLT-1 level.

## Discussion

 In this study, we aimed to investigate the effect of ceftriaxone, a beta lactam antibiotic, in VPA-induced mouse model of autism. Autism-like abnormal behavior was induced with a single injection (600 mg/kg) of VPA on the 12.5th day of gestation. Increased doses of ceftriaxone treatment had no effect on autism-like abnormal behavior. GLT-1 levels were increased in 200 mg/kg ceftriaxone groups in intrauterine saline exposed offsprings, but this increase did not lead to any behavioral changes.

Autism is a common neurodevelopmental disease; however, the pathophysiological process leading to autism is not clear. Abnormalities in many neurotransmitters such as GABA and glutamate have been observed in ASD, and these results have been directly associated with the pathogenesis of ASD. Plasma GABA concentration was higher and glutamate/GABA ratio was lower in autistic individuals compared to normal.^10^ In contrast, other studies have shown a decrease in GABA and GABA/glutamate ratios in frontal lobe imaging in autistic individuals.^11^ Zhang et al^18^ declared that bumetanide treatment caused a decrease in the symptoms of individuals with autism by decreasing the GABA/glutamate ratio. Today, there are two theories that explain the relationship between autism and glutamate. The first theory is introduced by Carlsson in 1998 and suggests that autism is a hypoglutamatergic disease. Carlsson attributed this theory to the presence of autism-like symptoms in healthy people who were treated with NMDA antagonists and to the detection of brain damage in glutamate-rich neurons in autism.^19^ According to the second theory, autism is a hyperglutamatergic disease.^20^ Fatemi et al^16^ explained his theory with three findings; serum glutamate levels of individuals with autism are higher than normal individuals^21^; the glutamic acid decarboxylase enzyme, which is responsible for the destruction of glutamate, is reduced in the brain tissues of autistics and glial fibrillary acidic protein, a marker of gliosis in the brain tissues and astroglial-microglial activation, levels are higher in autistic individuals. Astroglia take glutamate from both the extracellular domain and synthesize glutamate from glutamine.^20,22^

Glutamate is a very important neurotransmitter for regulation of behavior; it is released from synaptic terminals and removed by glutamate transporters, especially GLT-1, in the hippocampus.^23^ GLT-1 plays a key role in regulation of glutamate concentration and disorders of its amount and function can cause abnormal behaviors.^24^ Recent studies have shown that chronic use of ceftriaxone, a beta-lactam antibiotic, increases the expression of GLT-1 depending on the dose.^25^ The inducing effect of ceftriaxone on GLT-1 expression has also been shown by our group.^15^ Moreover many β-lactam antibiotics, such as ampicillin, cefazolin, and cefoperazone exert increase in GLT-1 expression and enhance its function.^26^

In the present study, we predicted that if autism is a hyperglutamatergic disease; increased GLT-1 levels, induced by ceftriaxone, can ameliorate the behavioral symptoms of autism. However, we showed that GLT-1 levels was higher in VPA exposed offsprings without ceftriaxone treatment. Studies indicate that VPA increases the mRNA of EATTs in astrocytes and oligodendrocytes. VPA makes this effect via inhibition of histone deacetylase enzyme and increases the expression of not only GLT-1 but also other glutamate transporters.^27^ VPA increases GLT-1 expression in the hippocampus and cortex but decreases the expression in the cerebellum.^28^ There were increased GLT-1 levels both in VPA exposed offspring and saline treated groups in WB. We showed for the first time that VPA applied in the intrauterine period may lead to increase in GLT-1 levels in the postnatal period. Postnatal ceftriaxone treatment on day 47-55 did not increase GLT-1 levels in VPA-induced autistic mice; however, GLT-1 levels were increased in saline exposed 200 mg/kg ceftriaxone treated group in WB. This increase may also be due to elevation in GLT-1 levels by VPA exposure in the prenatal period.

In this study, chronic ceftriaxone treatment did not exert any effect in VPA exposed offspring. There is no research on the effect of ceftriaxone in autism model of animals; however, a case report reported that beta lactam, cefixime, treatment reduced aggressive behaviors in an autistic child.^29^ Although the improvement in aggressive behavior was explained by the drug interaction of beta cefixime, which increases the antiepileptic drug level.^30^ This case report was one of the important reasons for starting this research project.

In our study, dihydrokainic acid, a GLT-1 inhibitor, was administered to evaluate whether ceftriaxone effect was related with GLT-1. In the first part of 3 CHM test, sniffing time of the empty/full cage was decreased when dihydrokainic acid was administered alone and in combination with ceftriaxone (200 mg/kg); this effect can be explained as decreased sociability. However, there was a decrease in sniffing time of the unfamiliar animal in the combination group in the second part of 3 CHM, but there was no increase in sniffing time of the familiar animals; this effect can also be speculated as decrease in sociability. It was reported that dihydrokainic acid injection into the central nucleus of amygdala elicited increased anxiety and depressive behavior in normal rats,^31^ but there is no research yet on effects of dihydrokainic acid in autism.

 In our study, GLT-1 expression was evaluated using WB and IHC analysis. In WB, there was a significant increase in GLT-1 levels in VPA exposed offspring. Recent research indicates that VPA increase GLT-1 levels due to its epigenetic effects. However, no study examined the effect of a single dose of VPA administered in the intrauterine period on GLT-1 levels in the postnatal period. Our research showed that the enhancing effect of VPA on GLT-1 levels in the intrauterine period continued in the postnatal period; however, we did not observe this increase in IHC analysis. It is remarkable that there is no correlation in the results of WB and IHC. The difference between these two analyses may be due to the difference between sampling methods. Punch biopsy was taken randomly from the hippocampus for WB analysis, whereas IHC was performed separately in different regions of the hippocampus. In addition, four out of eight animals in each group were allocated to the right and the other four to the left hemispheres for WB and IHC. The difference in outcome may be due to differences in right and left hemispheres in autism. Concerning our future research our expectation is that removing hippocampus totally and performing analyses in the same hemisphere will ensure the reliability of our results.

 Two theories can be developed by evaluating the results of our study. First, if VPA injection during the intrauterine period causes autism-like behavior because of the increase in GLT-1 levels, it can be a hypoglutamatergic disease. This is because increased GLT-1 will lead to decrease in glutamate concentration in the synaptic cleft. However, the question that should be kept in mind is whether the effect a single VPA exposure during the intrauterine period will continue for so long. The second theory is that autism is a hyperglutamatergic disease and GLT-1 levels increase as a compensation mechanism. If autism is a hyperglutamatergic disease, drugs that increase GLT-1 levels such as ceftriaxone may provide a hope for treatment of autism. However, to support these theories, studies on different autism models are needed.

## Conclusion

 Our results show that ceftriaxone did not exert significant therapeutic effect on VPA-induced mouse model of autism. But our study has some limitations so different studies are needed.

## Acknowledgments

 We thank Ahmet Ulugol, Çetin Hakan Karadag, K. Duvan-Aydemir and Onur Ersoy for their support. This work was supported by a grant from Trakya University Research Council (TUBAP-2017/190).

## Ethical Issues

 The experimental protocol of this study was approved by the local ethics committee. (Protocol no: TUHADYEK-2016/43).

## Conflict of Interest

 The authors have no conflicts of interest to declare.
